# Psychopharmacological management in patients with Di George syndrome

**DOI:** 10.1192/j.eurpsy.2024.944

**Published:** 2024-08-27

**Authors:** L. Rojas Vázquez, P. Marqués Cabezas, G. Lorenzo Chapatte, M. Ríos Vaquero, A. Monllor Lazarraga, M. P. Pando Fernández, M. A. Andreo Vidal, M. Calvo Valcárcel, P. Martínez Gimeno, M. J. Mateos Sexmero, B. Rodríguez Rodríguez, M. Fernández Lozano, N. Navarro Barriga, T. Jiménez Aparicio, C. De Andrés Lobo, C. Vallecillo Adame, L. Sobrino Conde, M. D. L. Á. Guillén Soto, A. Aparicio Parra

**Affiliations:** Psiquiatría, Sacyl - Hospital Clínico Universitario Valladolid, Valladolid, Spain

## Abstract

**Introduction:**

It is widely described in the scientific literature that patients who suffer from some type of congenital syndrome such as Di George Syndrome are more likely to present some type of psychopathological alteration during their development that may require intervention and treatment by infant and juvenile mental health teams in coordination with neuropediatrics (1). On this occasion, we will present the clinical case of a patient who regularly attends psychiatry consultations for management of anxious symptoms with impulse control deficits associated with intellectual disability, diagnosed since childhood with tetralogy of Fallot and later with Di George syndrome. In this type of case, treatment is usually considered taking into account possible comorbidities at the organic level (since there may be cardiological involvement, which can be an added difficulty when taking into account the adverse effects of some psychotropic drugs) (2).

**Objectives:**

This is followed by the presentation of the clinical case, which can serve to exemplify this type of case and clarify any doubts that may arise regarding treatment.

**Methods:**

Presentation of the clinical case and review of updated scientific literature on the subject.

**Results:**

Patient who first came to the infantile-junior consultations at the age of 8 years due to delay in the acquisition of verbal language and impulsivity. The patient had a history of pediatric follow-up since birth for different physical symptoms that finally led to the diagnosis of Di George syndrome.

Given the difficulties he presented both at home and at school, different psychometric tests were performed and it was determined that it could be beneficial to initiate treatment with extended-release methylphenidate. Prior to treatment, psychomotor restlessness (without aggressiveness) and difficulty in concentration prevailed, which improved significantly after upward adjustment of the dose to a guideline corresponding to his age and weight. It was not necessary in this case to administer other treatments (the possibility of starting Aripiprazole in case of episodes of agitation was considered, but it was not necessary). The patient has continued to be monitored by cardiology to assess the possible side effects of the treatment (since it can increase heart rate and blood pressure (3), but so far no complications have been detected).

Thanks to psychotherapeutic and educational intervention, language acquisition was achieved, although to date he still requires support due to the difficulties he still presents.

**Conclusions:**

It is important to take into account the possible side effects of psychopharmacological treatment in patients with an associated congenital syndrome. Intensive and comprehensive follow-up by psychiatry and pediatrics (and later by their primary care physician) should be performed.

**Disclosure of Interest:**

None Declared

